# Disclosure to sexual partner and condom use among HIV positive clients attending ART clinic at a tertiary health facility in South West Nigeria

**DOI:** 10.11604/pamj.2014.18.245.4371

**Published:** 2014-07-25

**Authors:** Ayodeji Matthew Adebayo, Olayinka Stephen Ilesanmi, Bridget Ama Omotoso, Oladele Olufemi Ayodeji, Adesola Olawumi Kareem, Faith Osaretin Alele

**Affiliations:** 1 Department of Community Medicine, University College Hospital, Ibadan, Nigeria; 2 Department of Preventive and Primary Care, Faculty of Public Health, College of Medicine, University of Ibadan, Oyo State, Nigeria; 3 Department of Community Health, Federal Medical Centre, Owo, Ondo State, Nigeria

**Keywords:** Condom, disclosure, sex, HIV, Nigeria

## Abstract

**Introduction:**

Condom use and disclosure of HIV status increase the safety of sexual activity. Its extent will determine the need for appropriate interventions. The objective of this study was to identify determinants of condom use and disclosure to sexual partners among individuals receiving Antiretroviral Therapy at a tertiary health facility in South West Nigeria.

**Methods:**

A cross-sectional study of 578 clients enrolled in the ART program of Federal Medical Centre Owo, Ondo State Nigeria, was conducted.

**Results:**

The mean age of respondents was 38.6+9.6 years, more than half (66.6%) were females and 7% were currently married. Three-quarter were sexually active out of which 324(75.9%) used condom consistently and correctly and 323(75.6%) disclosed their status to their sexual partner. Use of condom was by 81% of those with tertiary education (p=0.002), and 84.5% of singles utilized condom (p<0.001). Determinant of condom use wwere, male (OR: 2; CI: 1.1- 3.3; p=0.013), secondary and tertiary education (OR: 3.69; CI: 1.48 - 9.19; p=0.005) and (OR: 4.79; CI: 1.84 - 12.44; p=0.001) respectively. Determinant of disclosure was being married (OR: 11.8; CI- 5.5-25.7; p<0.001). No significant association exist between disclosure and condom use.

**Conclusion:**

Most of the people living with HIV accessing ART were sexually active. A good proportion of them used condom consistently and correctly. Disclosure did not have significant effect on condom use. More health education intervention to increase disclosure rate and safe sexual behaviour among HIV positive clients is needed.

## Introduction

Human immunodeficiency virus infection (HIV) and acquired immunodeficiency syndrome (AIDS) epidemic has become a serious developmental and health problem in many countries around the world [1]. Globally, an estimated 35.3 (32.2-38.8) million people were living with HIV in 2012 [2]. HIV/AIDS is ravaging sub-Saharan Africa, with greater than 60% of all HIV infections in the world occurring in this Region [3]. AIDS continues to be the leading cause of death in Africa, the prevalence in Nigeria is 4.6% and studies have shown that condoms are highly effective in preventing transmission when used correctly and consistently [4, 5]. HIV and AIDS is to a large extent a crisis of sexual behaviour, as unsafe sex is responsible for majority of HIV infection in the sub-Saharan Africa [5].

HIV counselling and testing programmes emphasis is placed on the importance of HIV status disclosure among HIV-infected clients, particularly to their sexual partners. Disclosure may motivate sexual partners to seek testing, change behaviour and ultimately decrease transmission of HIV. In addition, other health behaviours that may improve the outcome of care of HIV positive clients are facilitated [6].

Morbidity and mortality rates among HIV infected patients have decreased as a result of the introduction of antiretroviral therapy (ART), more people with HIV live longer and healthier lives [7]. Condom use by sero-concordant couples may be necessary not only to prevent pregnancy and STIs but also HIV drug-resistant and super-infection [8]. Therefore, the non-use of condom by sero-concordant couples encourages the spread of resistant strains of the virus and occurrence of super-infection, and the non-disclosure of status usually has a psychosocial effects on clients [9].

To further reduce the transmission of HIV/AIDS infection and for positive clients to live a healthier and more productive lives, news on disclosure and condom use should be spread widely. Increasing the level of disclosure of HIV status and decreasing risky sexual activities will not be effective if its extent is not known. This study aimed at assessing the magnitude of disclosure to sexual partners and condom use among HIV positive client and the effect of disclosure on condom use among clients attending ART clinic, FMC Owo

## Methods

### The Study Area

The study was conducted in Federal Medical Centre, Owo. It is located in the Owo Local Government Area of Ondo state in South West Nigeria, at the intersection of roads from Akure, kabba and Benin City. The hospital provides healthcare services at the primary, secondary and tertiary levels to the people within its catchment areas which are Ondo, Kogi, Edo, Ekiti and Osun States and its surrounding states. It also receives patients from almost all the states of the Federation because it is situated a stone throw from the highway that links Abuja to Lagos. It is also an approved training centre by both the West African Postgraduate College and the National Postgraduate College to train Resident Doctors in some specialist area of Medicine. Presently the centre has 21 clinical and 7 non-clinical departments [10]. It is a 250 bed tertiary health centre with average monthly attendance, by all age groups, at the outpatient department put at about 4,980 and the bed occupancy of not less than 70% at every point in time. The staff strength of the hospital is above 1000 [10].

### Study Instrument

The study was carried out in the ART clinic of FMC, Owo. A descriptive cross sectional design was used. In all, 578 consenting adults on HAART who attended the clinic over 3 months were recruited. Appropriate sample size was calculated using the Leslie Kish formula. Data was analysed with SPSS version 21.0. Association between categorical variables were explored with Chi square test and determinants of disclosure to sexual partners and condom use were identified using logistic regression analysis at 5% level of significance.

Data quality was maintained through careful design of study tool. We used semi-structured interviewer administered questionnaire. Four medical officers at the department of community health were engaged as interviewers. Prior to the data administration, they were trained in ethics in HIV research and techniques of questionnaire administration. Pre-testing was done on 20 individuals not included in the study. The independent variables used include socio-demographic (age, sex, educational status, marital status, religion, occupation), Medical factors (clinical stage at the commencement of HAART, duration on HAART), partners related factors (spouse status, current number of partner(s), disclosure of status to partner(s)), and condom related factors (condom use, sexual exposure). Multiple response questions were asked on the reasons for use and non-use of condom as the case maybe.

### Ethical Consideration

Ethical clearance was obtained from Health Research Ethics Committee of FMC Owo. Informed consent was obtained from respondents after the objective of the study was explained to them. Each respondent was given information on the benefit of the study and that she/he could participate voluntarily and had the right to withdraw at any time without any negative effect.

## Results

The mean age of respondents was 38.6±9.6 years. Out of the 578 respondents 193(33.4%) were males and about half were married 339 (58.6%); 175(30.3%) of the respondents had tertiary education. The socio-demographic characteristic of the participants are as shown in Table 1.

**Table 1:** Sociodemographic characteristics of respondents

Variables	Frequency	Percent
**Sex**		
Male	193	33.4
Female	385	66.6
**Education**		
No Formal Education	41	7.1
Primary	151	26.1
Secondary	211	36.5
Tertiary	175	30.3
**Marital Status**		
Married	339	58.7
Single	92	15.9
Widow/Widower	79	13.7
Separated	51	8.8
Divorced	17	2.9
**Religion**		
Christianity	546	94.5
Islam	32	5.5
**Occupation**		
Trading	214	37.0
Student/Unemployed	127	22
Civil Servant	123	21.3
Artisan	71	12.3
Farming	43	7.4

**Figure 1:**
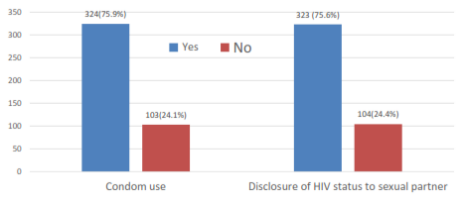
Proportion of sexually active respondents who used condom consistently and disclosed HIV status to sexual partner

Figure 1 shows the proportion of sexually active respondents who used condom consistently and the proportion of sexually active respondents who disclosed HIV status to sexual partner. In all, 427 (73.9%) of the clients had been sexually active since they were diagnosed of HIV. Consistent and correct condom use was reported by 324 (75.9%) of the clients. About three quarter 323 (75.6%) disclosed their HIV status to sexual partner.

Table 2 shows the association between socio-demographic characteristics and consistent and correct condom use. A significantly higher proportion 141(84.4%) of male respondents used condom consistently compared to 183(70.4%) of female. Among respondents who had tertiary education 98(81.0%) used condom consistently and correctly compared to 13(50%) of those without formal education (p=0.002). Also, 49(84.5%) of single respondents used condom consistently and correctly compared to 23(59.0%) widow/widower (p<0.001). No significant association exist between disclosure to sexual partner and use of condom though a slightly higher proportion 252(78.0%) of those who disclosed their HIV status used condom compared to 64(70.3%) who did not disclose.

**Table 2:** Association between sociodemographic characteristics and consistent and correct condom use

Socio-demographic Characteristics	Condom Use		Chi square	p value
	Yes	No		
**Age**				
Less than 25	8(66.7%)	4(33.3%)	2.660	0.447
25-34	126(78.3%)	35(21.7%)		
35-44	118(72.4%)	45(27.6%)		
45 and Above	72(79.1%)	19(20.7%)		
**Sex**				
Male	141(84.4%)	26(15.6%)	10.962	0.001
Female	183(70.4%)	77(29.6%)		
**Educational Status**				
No Formal Education	13(50%)	13(50%)	10.026	0.002
Primary Education	85(71.4%)	34(28.6%)		
Secondary Education	128(79.5%)	33(20.5%)		
Tertiary Education	98(81.0%)	23(19.0%)		
**Marital Status**				
Single	49(84.5%)	9(15.5%)	20.588	<0.001
Married	228(77.8%)	65(22.2%)		
Separated	21(77.8%)	6(22.2%)		
Divorced	3(30%)	7(70%)		
Widow/Widower	23(59.0%)	16(41.0%)		
**Religion**				
Christianity	308(76.2%)	96(23.8%)	0.529	0.467
Islam	16(69.6%)	7(30.4%)		
**Occupation**				
Civil Servant	66(74.2%)	23(25.8%)	5.862	0.210
Trading	115(73.7%)	41(26.3%)		
Farming	21(70%)	9(30%)		
Artisan	42(72.4%)	16(27.6%)		
Student/Unemployed	80(85.1%)	14(14.9%)		
**Spouse/Partner Status**				
Positive	123(75.5%)	40(24.5%)	1.223	0.546
Negative	121(78.6%)	33(21.4%)		
I don't know	80(72.7%)	30(27.3%)		
**Number Current Sexual Partner**				
1	306(75.2%)	101(24.8%)	2.286	0.131
>1	18(90%)	2(10%)		
**Disclosure to Sexual Partner**				
Yes	252(78.0%)	71(22.0%)	2.323	0.127
No	64(70.3%)	27(29.7%)		

In all, 260(88.7%) of married respondents disclosed their status to their partners compared to 29(50%) of single respondents (p<0.001). Other association between socio-demographic characteristics and disclosure of HIV status to sexual partner is as shown in Table 3.

**Table 3:** Association between sociodemographic and other characteristics with disclosure of HIV status to sexual partner

Variables	Disclosure of HIV Status		Chi square	p value
	Yes	No		
**Age**				
Less than 25	8(66.7%)	4(33.3%)	2.137	0.544
25-34	117(72.7%)	44(27.3%)		
35-44	126(77.3%)	37(22.7%)		
45 and Above	72(79.1%)	19(20.9%)		
**Sex**				
Male	138(82.6%)	29(17.4%)	7.275	0.07
Female	185(71.2%)	75(28.8%)		
**Educational Status**				
No Formal Education	17(65.4%)	9(34.6%)	3.657	0.301
Primary	88(73.9%)	31(26.1%)		
Secondary	120(74.5%)	41(25.5%)		
Tertiary	98(81.0%)	23(19.0%)		
**Marital Status**				
Single	29(50%)	29(50%)	90.041	<0.001
Married	260(88.7%)	33(11.3%)		
Separated	15(55.6%)	12(44.4%)		
Divorced	4(40%)	6(60%)		
Widow/Widower	15(38.5%)	24(61.5%)		
**Occupation**				
Civil Servant	69(77.5%)	20(22.5%)	8.578	0.073
Trading	106(67.9%)	50(32.1%)		
Farming	25(83.3%)	5(16.7%)		
Artisan	46(79.3%)	12(20.7%)		
Student/Unemployed	77(81.9%)	17(18.1%)		
**Condom Use**				
Yes	71(68.9%)	32(31.1%)	3.319	0.068
No	252(77.8%)	72(22.2%)		
**Number Current Sexual Partner**				
1	309(75.9%)	98(24.1%)	0.363	0.547
>1	14(70.0%)	6(30.0%)		

Significantly, the odds of male respondents to have used condom is two times compared to female (OR: 2 (CI- 1.1- 3.3) p=0.013). Consistent and correct Condom use rate was higher in respondents who had formal education especially tertiary and secondary education (OR: 3.69 (CI- 1.48 - 9.19)) and (OR: 4.79(CI- 1.84 - 12.44)) respectively than those without formal education. The number of current sexual partner(s) did not significantly affect condom use. Table 4 shows the predictors of condom use among respondents.

**Table 4:** Predictors of condom use among respondents

Variables	Odds Ratio	95% CI	P Value
**Sex**			
Male	1.95	1.15-3.30	0.013
Female	1		
**Marital Status**			
Single	2.85	1.03 - 7.89	0.044
Married	1.74	0.78 - 3.86	0.173
Separated	1.89	0.59 - 6.15	0.286
Divorced	0.27	0.06 - 1.31	0.104
Widow/Widower	1		
**Educational Status**			
No Formal Education	1		
Primary	2.56	1.02 - 6.44	0.046
Secondary	3.69	1.48 - 9.19	0.005
Tertiary	4.79	1.84 - 12.44	0.001

Table 5 shows the predictors of HIV status disclosure to sexual partner. There was no statistical significance between the use of condom and disclosure of status to sexual partner partner (OR: 0.776; CI: 0.43-1.40; p=0.399). The odds of married couples disclosing their status was about 12 times more likely compared to sexually active widow/widower (OR: 11.8; CI- 5.5-25.7; p<0.001).

**Table 5:** Predictors of HIV status disclosure to sexual partner

Variables	Odds Ratio	Confidence Interval	P Value
**Sex**			
Male	1.141	0.64 – 2.04	0.656
Female	1		
**Marital Status**			
Single	1.273	0.531 - 3.052	0.589
Married	11.831	5.45 – 25.71	<0.001
Separated	1.960	0.70 – 5.49	0.200
Divorced	1.123	0.26 – 4.78	0.876
Widow/Widower	1		
**Condom Use**			
Yes	1		
No	0.776	0.43 – 1.40	0.399
**Occupation**			
Civil Servant	1		
Trading	0.540	0.268 – 1.086	0.084
Farming	0.961	0.283 – 3.267	0.949
Artisan	0.649	0.259 – 1.628	0.357
Student/Unemployed	1.280	0.551 – 2.975	0.566

## Discussion

Disclosure to sexual partner and condom use are vital in the prevention of the spread of HIV/AIDS infection, creating awareness among discordant couple and reduction in the HIV/AIDS pandemic [7]. This study is aimed at assessing the magnitude of disclosure to sexual partners and condom use among HIV positive client and identify contributory factors to condom use and disclosure of HIV status among the clients.

When used correctly and consistently, condoms remain one of the most efficient technologies available to prevent sexual transmission of HIV [2]. The results of the present study revealed about three-quarter of respondents that were sexually active used condom. A significantly higher proportion of male respondents used condom compared to female. Other Nigerian studies have reported same higher prevalence of condom use among males [11, 12]. One of the reasons for this may be due to the stigma of promiscuity attached to females suggesting the use of condom [13]. Inability of females to guarantee condom use if their partners did not wish to use it has been reported to be a reason for low condom use among females [12]. This study did not ascertain whether or not female condoms were readily available.

Condom use was significantly higher in singles than the married respondents. This is commendable since consistent and correct use of condom has been widely recommended as a public health strategy against sexually transmitted infections, including heterosexual transmission of HIV infection [14]. This finding is in contrast to the study done in Osun State, Nigeria which shows a higher prevalence among married respondents [15]. Desire for children could have prevented the married from using condom [16, 17]. The belief that witchcraft plays a role in HIV transmission has been shown to be related to less positive attitudes about condoms, less belief in condom effectiveness for HIV prevention, and lower intention to use condom among men [18]. Condom use in this study was higher in respondents who had formal education especially tertiary and secondary. Their awareness and knowledge about condom could have contributed to this as reported in another Nigerian study [11].

Disclosure of HIV status to sexual partner was reported by 75% of the respondents. In this study, the odds of married couples disclosing their status was about 12 times compared to singles as seen in most other studies reported in the developing countries [19, 20]. Though a study done in Abeokuta shows that low level of disclosure of HIV status may raise the possibility of not practising safer sex [21]. Women who disclosed their status to their partners are more likely to participate in the prevention of Mother to child transmission, hence fulfilling one of the goals of HIV/AIDS prevention as outlined by the Centre for Disease Control and Prevention (CDC) [22, 23]. Also studies have shown that disclosure improves adherence to ART treatment [24]. The effect of disclosure of HIV status on condom use in this study was not statistically significant though a higher proportion of respondents who disclosed their status used condom. This is raising a question - do clients understand what it means to prevent re-infection or super infection of HIV/AIDS?

## Conclusion

Condom use in this study and disclosure of HIV status to sexual partner was reported by three quarter of the respondents. Males, singles and the educated were more likely to use condom. Married couples are more likely to disclose their status to sexual partner. Disclosure did not have significant effect on condom use. More health education intervention to increase disclosure rate and safe sexual behaviour among HIV positive clients is needed. **Recommendation**: More health education intervention is required especially through adherence counselling of all HIV positive clients about the importance of disclosure and the use of condom especially its dual protection. It will reduce the scourge of HIV/AIDS and assist clients to effectively assess their ART and participate fully in PMTCT. **Limitation**: The study did not captured desire for pregnancy, this may be a key factor affecting the use of condom among the married.
